# Potent Therapeutic Insights From *Eryngium campestre*: Phytochemical Profiling and In Vitro Evaluation

**DOI:** 10.1002/fsn3.72250

**Published:** 2026-08-10

**Authors:** Kübra Seyhan, Gamze Akıllı, Pınar Baloglu, Ayşenur Özdemir Kanat, Nurdan Tugba Iscen, Giovanni Caprioli, Agnese Santanatoglia, Massimo Ricciutelli, Mehmet Cengiz Baloglu, Yasemin Celik Altunoglu, Muammer Bahsi, Milena Terzic, Gökhan Zengin

**Affiliations:** ^1^ Plantomics Research Laboratory, Department of Genetics and Bioengineering, Faculty of Engineering and Architecture Kastamonu University Kastamonu Türkiye; ^2^ Department of Biology, Faculty of Science Kastamonu University Kastamonu Türkiye; ^3^ Chemistry Interdisciplinary Project (CHip), School of Pharmacy University of Camerino Camerino Italy; ^4^ Department of Primary Education, Faculty of Education Firat University Elazığ Türkiye; ^5^ Faculty of Technology Novi Sad University of Novi Sad Novi Sad Serbia; ^6^ Department of Biology, Science Faculty Selcuk University Konya Türkiye

**Keywords:** anticancer, antimicrobial, antioxidant, anti‐quorum sensing, *Eryngium campestre*, phytochemicals

## Abstract

The investigation of the biological activities of plants utilized in traditional medicine to treat various illnesses is of great importance for their potential evaluation as pharmaceutical raw materials in modern medicine. In the present study, the anticancer, antimicrobial, and anti‐quorum‐sensing (anti‐QS) activities of ethyl acetate, ethanol, ethanol‐water, and water extracts prepared from the aerial parts of *Eryngium campestre* by maceration were tested. The biological content, antioxidant, and enzyme‐inhibitory capacity of the extracts were also investigated. *E. campestre* extracts possess a rich flavonoid and phenolic composition. The ethanol‐water extract had the highest total polyphenol content. 3‐hydroxybenzoic acid, 4‐hydroxybenzoic acid, vanillic acid, p‐coumaric acid, and ferulic acid were common and abundant across all extracts. The ethanol extract showed the highest enzyme‐inhibitory activity in acetylcholinesterase (AChE) and butyrylcholinesterase (BChE) tests with values of 2.68 and 3.17 mg GALAE/g, respectively. The anticancer activity results revealed that the lowest 50% inhibitory concentration (IC_50_) for cell viability in the MDA‐MB‐231 cell line was 215.2 μg/mL for the ethanol extract. For the HeLa cell line, the lowest IC_50_ value was defined as 151.7 μg/mL for the ethyl acetate extract. No antimicrobial effects were detected in the tested concentrations of the extracts against reference microbial strains. To determine anti‐QS activity, the inhibitory effect on violacein pigment production was investigated on the *Chromobacterium violaceum* CV026 mutant strain. No inhibition of pigment production was observed in the study. Based on the findings, *E. campestre* may have potential for further anticancer research and pharmacological studies.

## Graphical abstract

The phytochemical profiles and biological activity of ethyl acetate, ethanol, ethanol‐water, and water extracts of *Eryngium campestre* were assessed. *E. campestre* is a prospective source of multifunctional bioactive chemicals for food and nutraceutical uses, as demonstrated by the extracts' potential antioxidant, enzyme‐inhibitory, anticancer, antibacterial, and anti‐quorum‐sensing capabilities.

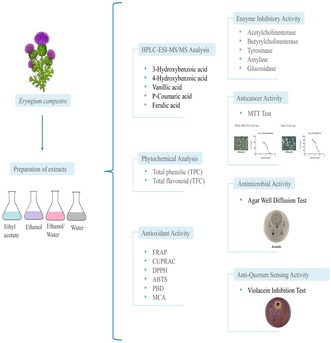

## 1 Introduction

*Eryngium* belongs to the Saniculoideae subfamily of the Apiaceae family, with 317 species. The primary growing regions for *Eryngium campestre* L. include central and southern Europe, northern Africa, and most of Asia (Güneş et al. 2014). In traditional Turkish medicine, *E. campestre* is known as “Bogadikeni” and occurs widely across Türkiye. *E. campestre* is a perennial plant with a thick, pale green, very spiky stalk that varies in length from 30 to 60 cm. Coriaceous basal leaves with limbs 5–20 cm long are evergreen. The white flowers have ovoid or globular crowns. The ovate fruits are covered with scales (Medbouhi et al. 2019).

Globally, several *Eryngium* species are cultivated and used as spices, while others are traditionally used to treat various diseases, including hypertension, gastrointestinal disorders, asthma, burns, fever, diarrhea, hypercholesterolemia, diabetes, and malaria (Cárdenas‐Valdovinos et al. 2023; Erdem et al. 2015). The pharmacological potential of *Eryngium* species is strongly associated with their rich phytochemical profile. Members of this genus contain a wide range of bioactive secondary metabolites, such as terpenoids, steroids, triterpenoid saponins, polyacetylenes, phenolic compounds, flavonoids, coumarins, and rosmarinic acid derivatives (Tel‐Çayan and Duru 2019).

*Eryngium campestre* is widely used in traditional folk medicine. Different plant parts, including leaves, flowers, stems, roots, and aerial parts, are employed for therapeutic purposes. The species is particularly used to treat urinary and kidney disorders, including kidney stones. It is also used for cough, bronchitis, inflammatory conditions, wounds, snake bites, and gynecological diseases. In addition, diuretic and aphrodisiac effects have been reported (Karaoğlan 2026). Phytochemical analyses of *E. campestre* have identified nine phenolic acids, namely chlorogenic, ferulic, 3,4‐dihydroxyphenylacetic, caffeic, protocatechuic, rosmarinic, syringic, 4‐feruloylquinic, and 4‐dihydroxybenzoic acids. Five flavonoids have also been detected, including kaempferol, quercitrin, rutin, quercetin, and astragalin (Al‐Askar et al. 2023).

Various enzymes play important roles in the development of many diseases that threaten human health, such as cancer, diabetes, and Alzheimer's disease (Pan and Kakeya 2025). Enzyme inhibitors, which bind to the target enzyme's active site, reduce its catalytic activity by blocking it. Enzyme inhibitors are used to target cancer cells and reduce their growth. For example, tyrosine kinase inhibitors block tyrosine kinase activity, which is involved in the proliferation and growth of cancer cells. In addition, enzyme inhibitors are also used in the treatment of bacterial and fungal infections. For example, β‐lactamase inhibitors are used to treat infections caused by certain bacterial strains (Singh et al. 2023). Therefore, enzyme inhibitors are widely used in research due to their therapeutic potential. Studies are investigating the enzyme inhibitory potential of *Eryngium* species. These effects are thought to be related to bioactive components in plants, such as phenolic compounds, flavonoids, and terpenoids (Aliyev and Abdullayeva 2025; Pérez‐Muñoz et al. 2022; Romo‐Pérez et al. 2022; Zengin et al. 2024).

Extracts from different *Eryngium* species have demonstrated various in vitro bioactivities. These include cytotoxic effects, anti‐inflammatory properties, venom‐neutralizing activity against snake venoms, and antibacterial, antifungal, antimalarial, antioxidant, and antihyperglycemic activities (Medbouhi et al. 2019; Price III 2016). Additionally, in vivo studies using multiple animal models have yielded encouraging results (Wang et al. 2012). The methanolic extract of aerial parts of *E. campestre* demonstrates potent antitumor activity against potato tumor cells stimulated by *Agrobacterium tumefaciens* (Usta et al. 2014). Aerial portions of *E. campestre* were used to extract essential oil, which contained several different compounds: eudesma‐4(15)‐7‐dien‐1‐ol, myrcene, α‐cadinol, spathulenol, germacrene D, campestrolide, and germacrene B. All of these substances have shown vigorous antibacterial activity against Gram‐positive bacteria. In particular, it has been found that plants in the Eastern Mediterranean basin produce kadinan, guaiane, and oxygenated compounds, which are found in essential oil‐producing plants (Al‐Askar et al. 2023; Medbouhi et al. 2019).

Medicinal plants are rich sources of secondary metabolites, including phenolics, flavonoids, alkaloids, lignans, and terpenoids, which are associated with various biological activities. The extraction efficiency of these compounds is strongly influenced by the type and polarity of the solvent used. Solvent polarity can affect the yield, phytochemical composition, antioxidant capacity, and biological activity of plant extracts (Wakeel et al. 2019). Therefore, ethyl acetate, ethanol, ethanol–water, and water were selected in the present study to obtain extracts with different phytochemical profiles and biological properties.

*Eryngium* species have been reported to exhibit notable anticancer activities against various cancer cell lines. In particular, *E. thyrsoideum* and *E. kotschyi* have demonstrated significant cytotoxic effects against MDA‐MB‐231 breast cancer cells, with ethyl acetate extracts showing the highest activity (Hosseini et al. 2022; Arslan et al. 2022). Similarly, *E. creticum* extracts have been shown to exert dose‐dependent antiproliferative effects on HeLa cervical cancer cells, with leaf extracts representing the most active fraction (Dirani et al. 2014). Antimicrobial activity has also been reported for *E. campestre* , although this effect appears to be strain‐dependent, with selective bacteriostatic activity particularly against *Pseudomonas aeruginosa* (Conea et al. 2016). However, most previous studies have focused on specific extracts and a limited number of biological endpoints, and comprehensive comparative evaluations of different solvent extracts remain scarce. In this context, the present study aimed to evaluate the ethyl acetate, ethanol, ethanol–water, and water extracts of *E. campestre* , investigating their antibacterial, antitumor, antioxidant, anti‐quorum sensing, and enzyme‐inhibitory activities using an integrated multi‐assay approach to provide a more comprehensive assessment of its biological potential.

## 2 Materials and Methods

### 2.1 Collection and Identification of Plant Material

In 2022, plant materials were gathered from the Elazığ (Sahinkaya village) province in Turkey. Dr. Ugur Cakilcioglu carefully conducted the botanical characterization of the collected specimens. A voucher sample was placed at Munzur University's herbarium for record‐keeping and future use (UC‐22‐16). The plant's aerial parts were meticulously separated after harvest and air‐dried at room temperature in a shady location to maintain their phytochemical profile. After drying, the material was ground into a fine powder using a consistent grinding procedure. To reduce deterioration and preserve long‐term stability, the powdered samples were then kept in containers that could withstand regulated environmental conditions.

### 2.2 Preparation of Crude Extracts

Ethyl acetate (EA), ethanol, ethanol‐water, and water were used to extract the bioactive components. 200 mL of each solvent was used to treat a typical 10‐g sample amount. Depending on the solvent, the procedure varied: the water‐based approach required a quick 15‐min hot‐water infusion, whereas organic extractions required a 24‐h maceration at ambient temperature. The aqueous extract was freeze‐dried for post‐processing (Syclon‐10 N (Ningbo Haishu Sklon Electronic Instrument Inc., Ningbo, China, −85°C)), and rotary evaporation (Laborota‐4000, Heidolph Instruments, Germany) was used to remove the organic solvents at reduced pressure. The extraction yield (%) was calculated using the following equation: Extraction yield (%) = (weight of dried extract/weight of dried plant material) × 100.

### 2.3 Assay for Total Phenolic and Flavonoid Content

Total phenolic and flavonoid contents in the extracts were measured using previously established procedures (Zengin and Aktumsek 2014). Total phenolic content and total flavonoid content were expressed as gallic acid equivalents (GAE, mg GAE/g dry extract) and rutin equivalents (RE, mg RE/g dry extract), using gallic acid (GA) and rutin (R) as standards. Triplicate measurements were taken for these experiments. Data were analyzed using one‐way ANOVA (GraphPad Prism, version 9.2), followed by Tukey's post hoc test for pairwise group comparisons. A *p*‐value below 0.05 was regarded as statistically significant.

### 2.4 Assays for In Vitro Antioxidant Capacity

Antioxidant assessments were performed according to recognized protocols, as detailed in an earlier study (Grochowski et al. 2017). Numerous assays, such as ferric reducing antioxidant power (FRAP), cupric reducing antioxidant capacity (CUPRAC), 2,2‐Diphenyl‐1‐picrylhydrazyl (DPPH), and 2,2′‐azinobis (3‐ethylbenzothiazoline‐6‐sulfonic acid) (ABTS), were used to assess the extracts' potential to scavenge radicals. Milligrams of trolox equivalents (TE) per gram of dried extract were used to express the results. The phosphomolybdenum (PBD) test was then used to calculate the total antioxidant potential, expressed as mmol TE/g. Additionally, milligrams of EDTA equivalents (EDTAE) per gram of extract were used to quantify metal chelation activity (MCA). Triplicate measurements were taken for these experiments. Data were analyzed using one‐way ANOVA (GraphPad Prism, version 9.2), followed by Tukey's post hoc test for pairwise group comparisons. A *p*‐value below 0.05 was regarded as statistically significant.

### 2.5 Inhibitory Properties Against Some Key Enzymes

The evaluated extracts were used in enzyme inhibition experiments against acetylcholinesterase (AChE), butyrylcholinesterase (BChE), tyrosinase, amylase, and glucosidase using approved techniques documented in the literature (Grochowski et al. 2017). Milligrams of galanthamine equivalents (GALAE) per gram of dry extract were then used to measure the inhibitory actions toward AChE and BChE. Tyrosinase inhibition was measured in kojic acid equivalents (KAE) per gram of dry extract, while the inhibitory potential of amylase and glucosidase was represented in acarbose equivalents (ACAE). Triplicate measurements were taken for these experiments. Data were analyzed using one‐way ANOVA (GraphPad Prism, version 9.2), followed by Tukey's post hoc test for pairwise group comparisons. A *p*‐value below 0.05 was regarded as statistically significant.

### 2.6 HPLC‐ESI‐MS/MS Analysis

#### 2.6.1 Reagents and Standards

Cyanidin‐3‐glucoside chloride, delphinidin‐3,5‐diglucoside chloride, delphinidin‐3‐galactoside chloride, petunidin‐3‐glucoside chloride, malvidin‐3‐galactoside chloride, quercetin‐3‐glucoside, and kaempferol‐3‐glucoside were bought from PhytoLab (Vestenbergsgreuth, Germany). Sigma‐Aldrich (Milan, Italy) provided the other 31 analytical standards for the 38 phenolic ingredients.

With the exception of anthocyanins, which were kept at −15°C until analysis, pure standards were dissolved in HPLC‐grade methanol to create individual stock solutions of each analyte at 1000 mg L^−1^, which were then maintained in glass‐stoppered vials at 4°C. HPLC‐grade methanol was used to dilute the stock solutions to create standard working solutions at different concentrations every day. 99% formic acid was purchased from Merck (Darmstadt, Germany). Carlo Erba Reagents in Milan, Italy, provided the 37% analytical‐grade hydrochloric acid. HPLC‐grade methanol was provided by Sigma‐Aldrich (Milano, Italy). Deionized water (> 18 MΩ cm resistivity) was further purified using a Milli‐Q SP Reagent Water System (Millipore, Bedford, MA, USA). A Sartorius Stedim (Goettingen, Germany) 0.2 μm polyamide filter was used to filter all solvents and solutions. A PhenexTM RC 4 mm 0.2 μm syringeless filter (Phenomenex, Castel Maggiore, BO, Italy) was used to filter all samples prior to HPLC analysis.

#### 2.6.2 HPLC‐ESI‐MS/MS Conditions

An Agilent 1290 Infinity series and a Triple Quadrupole 6420 from Agilent Technology (Santa Clara, CA) with an electrospray ionization (ESI) source that operates in both positive and negative ionization modes were used for HPLC–MS/MS investigations. Actually, the device allowed a single polarity‐switching run without any issues. Each analyte's MS/MS parameters were adjusted in flow injection analysis (FIA) using Optimizer Software (Agilent) (1 μL of a 10 mg L^−1^ individual standard solution). A Synergi Polar–RP C18 analytical column (250 × 4.6 mm, 4 μm) from Phenomenex (Chesire, UK) was used to separate the target chemicals. Prior to the column was the Polar RP security guard cartridge (4 × 3 mm ID). In gradient elution mode, the mobile phase consisted of a combination of (A) water and (B) methanol with 0.1% formic acid at a flow rate of 0.8 mL min^−1^. The ingredients of the mobile phase changed over time: 0–1 min, isocratic condition, 20% B; 1–25 min, 20%–85% B; 25–26 min, isocratic condition, 85% B; 26–32 min, 85%–20% B. A 0.2 μm polyamide filter from Sartorius Stedim (Goettingen, Germany) was used to filter all solvents and solutions. Two microliters was the injection volume. The ionization source's drying gas temperature was 350°C, whereas the column temperature was 30°C. A 55 psi nebulizer pressure, a 4000 V capillary voltage, and a 12 L/min flow rate were used. The dynamic‐multiple reaction monitoring (dynamic‐MRM) mode was used for detection, and the dynamic‐MRM peak regions were combined for quantification. Quantitation was done using the most prevalent product ion, and qualifying was done using the others. Each compound's unique time window (Δ retention time) was positioned at two minutes.

### 2.7 Anticancer Activity Test

#### 2.7.1 Cancer Cells and Growth Conditions

MDA‐MB‐231 and HeLa cell lines were sourced from the Department of Molecular Biology and Genetics at Boğaziçi University for this study. MDA‐MB‐231 is a human breast adenocarcinoma cell line classified as triple‐negative breast cancer (TNBC) due to the absence of progesterone (PR), estrogen (ER), and human epidermal growth factor (HER‐2) receptors. HeLa cells are a cell line derived from cervical cancer. The MDA‐MB‐231 cell line was cultivated in Dulbecco Modified Eagle's Media (DMEM, Gibco high glucose, Thermo Fisher Scientific, USA). The HeLa cell line was cultured in DMEM (Euroclone high glucose, Milan, Italy). Both media contained 10% Fetal Bovine Serum (FBS, Gibco, Thermo Fisher, USA), 1% Penicillin/Streptomycin (Pen‐Strep, Gibco, Thermo Fisher, USA), and Non‐Essential Amino Acids (NEAA, Pan biotech MEM 100X, Germany) and were incubated in 100 mm sterile Petri dishes at 37°C, 5% CO_2_ conditions.

#### 2.7.2 Preparation of Plant Extracts for Cell Culture Experiments

To assess their in vitro cell viability, stock solutions of EA, ethanol, ethanol‐water, and water extracts of *E. campestre* prepared by the maceration technique were formulated at a final concentration of 20 mg/mL. Plant extracts were dissolved in sterile ultrapure water and/or DMSO (Dimethyl sulfoxide, Isolab, Biofroxx) to obtain stock solutions. Each working stock was prepared at varying concentrations (1000, 500, 250, 125, 62.5, and 31.25 μg/mL) by serial dilution from the base stock. All prepared extract solutions were stored at −20°C.

#### 2.7.3 MTT Test for Cell Viability Detection

The effects of the extracts on cell lines, specifically their antiproliferative properties, were examined using the MTT (3‐(4,5‐dimethylthiazol‐2‐yl)‐2,5‐diphenyltetrazolium bromide) test (Sharif et al. 2021). Cells in the logarithmic phase of culture were harvested from Petri dishes, and cell counts were performed using a Thoma slide. MDA‐MB‐231 and HeLa cells were subsequently seeded in 96 well plates with 10,000 cells per well. After the cells reached 90% confluence, EA, ethanol, ethanol‐water, and water extracts were incubated for 48 h at several concentrations (31.25, 62.5, 125, 250, 500, and 1000 μg/mL) in 100 μL of complete growth medium. For the control groups, 10 μL of ultrapure water was used as the solvent for the extracts. The cells were then imaged under an inverted microscope at 100× magnification at the end of the incubation periods. After aspirating the cell media, 100 μL of MTT solution (0.5% FBS in DMEM and 0.5 mg/mL MTT) was added. The cells were then incubated for 4 h at 37°C and 5% CO_2_. Following incubation, the contents of the wells were discarded, and the subsequent formazan crystals were dissolved in pure DMSO (100 μL). Using a spectrophotometer (Multiscan GO, Thermo Scientific, USA), the absorbance at 570 nm was measured. Results were expressed as a percentage (%) by comparing cell viability in the extract‐treated wells with that in the control wells. The half‐maximal inhibitory concentration (IC_50_) values were calculated using GraphPad Prism 8. The experiments were independently repeated three times. The data were presented as mean ± standard deviation, and *p*‐values < 0.05 indicated statistical significance.

### 2.8 Antibacterial Activity Analysis

To determine the antibacterial effect of the extracts, three Gram‐positive ( *Bacillus subtilis* ATCC 6633, *Staphylococcus aureus* ATCC 25923, and *Enterococcus faecalis* ATCC 2921) and six Gram‐negative bacterial [*Enterobacter (Klebsiella) aerogenes* ATCC13048, *Acinetobacter haemolyticus* ATCC 19002, *Pseudomonas aeruginosa* ATCC 27853, *Salmonella Typhimurium* ATCC 14028, *Klebsiella pneumoniae* ATCC 13883, and *Escherichia coli* ATCC 25922] strains obtained from the Department of Genetics and Bioengineering at Kastamonu University were used.

#### 2.8.1 Agar Well Diffusion Test

Antimicrobial activity was assessed using the agar well diffusion method, following the guidelines of the European Committee on Antimicrobial Susceptibility Testing (EUCAST) (EUCAST 2025). Briefly, overnight cultures of each strain grown on agar medium were prepared as a suspension of colonies at a 0.5 McFarland density. Streaking was performed in three directions on Mueller‐Hinton Agar (MHA) utilizing sterile cotton swabs for inoculation. Subsequently, 6‐mm‐diameter wells were punched into the blank discs, and 50 μL of the 20 mg/mL working stock of the extract (1000 μg/well) was added to each well. Levofloxacin (5 μg/well) was used as the reference antimicrobial agent in the experiments. The solvents used in the extracts served as negative controls. A ruler was used to measure the widths of the inhibition zones (ZOI) around the wells (mm) after 24 h of growth at 37°C.

### 2.9 Quorum‐Sensing (QS) Inhibition Tests

#### 2.9.1 Violacein Inhibition Test

The McClean et al. (1997) method for examining the extract's ability to prevent violacin pigment synthesis used the bioindicator strain *Chromobacterium violaceum* CV026 in the soft agar method. In summary, 50 μL of *C. violaceum* in LB broth was added to 5 mL of melted soft LB agar (0.5% w/v) and incubated for the whole night at 37°C and 150 rpm. After incubation, 50 μL of the culture was used to inoculate 5 mL of melted LB agar (0.5% agar). The soft agar culture mixture was vortexed and poured directly onto pre‐prepared LB agar plates. Six‐millimeter diameter wells were cut into the frozen agar for the extracts. The extract (20 mg/mL) was pipetted into the wells. Fifty microliters of the supernatant from an overnight culture of *C. violaceum* ATCC 12472 strain was used as a positive control, while the solvent of the extracts was used as a negative control. The cultures were incubated at 37°C for 24 h, then analyzed for violacein production. Quorum‐sensing (QS) inhibition was determined by the presence of a colorless, opaque, frosted glass‐like zone surrounding the wells.

## 3 Results and Discussion

### 3.1 Biological Content of *E. campestre*

The bioactive properties of plant‐derived phenolic compounds and flavonoids have attracted significant attention in recent years in the pharmaceutical, food, and cosmetic industries. These compounds exhibit various biological activities, particularly antioxidant, anticancer, antimicrobial, and anti‐inflammatory effects (Daglia 2012; Panche et al. 2016).

Phytochemical studies on *E. campestre* have identified various bioactive compounds, including flavonoids, coumarins, monoterpene glycosides, saponins, phenolic acids, and essential oils. Flavonoids are mainly found in the aerial parts, whereas coumarins, monoterpene glycosides, and saponins are primarily present in the roots (Kartal et al. 2005, 2006; Nebija et al. 2009).

The results showed that the largest amount of total polyphenols was found in the ethanol‐water extract. In this extract, significant amounts of important phenolic compounds, including Kaempferol‐3‐glucoside, Chlorogenic acid, Ferulic acid, 3‐Hydroxybenzoic acid, 4‐Hydroxybenzoic acid, Caffeic acid, and p‐Coumaric acid, were identified. Some of these compounds were also detected at noteworthy levels in the other extracts. Notably, 3‐Hydroxybenzoic acid, 4‐Hydroxybenzoic acid, Vanillic acid, p‐Coumaric acid, and Ferulic acid were found to be common and abundant across all extracts (Table 1). This finding demonstrates that *E. campestre* is rich in phenolic content, suggesting it may possess strong antioxidant capacity.

**TABLE 1:** Chemical components levels of *E. campestre* extracts obtained with different solvents.

Compounds	EA	Ethanol	Ethanol‐water	Water
Phenolic acids				
Hydroxybenzoic acids				
Gallic acid	1.92	8.91	173.72	64.82
4‐Hydroxybenzoic acid	776.37	1033.25	1274.83	3289.35
3‐Hydroxybenzoic acid	1312.37	1752.22	2204.16	5559.07
Vanillic acid	838.78	647.04	942.64	1046.56
Syringic acid	291.99	334.65	576.45	597.22
Ellagic acid	61.14	38.61	n.d.	n.d.
Hydroxycinnamic acids				
Neochlorogenic acid	0.14	9.18	317.86	104.58
Chlorogenic acid	13.52	367.22	3004.98	83.15
Caffeic acid	439.92	648.19	1151.33	219.27
p‐Coumaric acid	569.09	563.71	1035.77	615.84
Ferulic acid	1808.89	1313.98	2376.36	955.49
3,5‐Dicaffeoylquinic acid	0.40	11.65	23.87	n.d.
Trans‐cinnamic acid	n.d.	n.d.	24.09	20.98
Flavonoids				
Flavan‐3‐ols				
Catechin	n.d.	n.d.	n.d.	n.d.
Epicatechin	n.d.	n.d.	n.d.	n.d.
Proanthocyanidins (condensed tannins)				
Procyanidin A2	3.38	n.d.	n.d.	n.d.
Procyanidin B2	n.d.	n.d.	n.d.	n.d.
Flavonols				
Quercetin	15.12	24.21	n.d.	n.d.
Kaempferol	133.32	287.94	969.86	1597.83
Myricetin	n.d.	n.d.	n.d.	n.d.
Isorhamnetin	21.65	24.19	2.89	4.59
Rutin	8.78	384.33	845.56	3.06
Hyperoside	1.75	362.87	661.59	5.14
Isoquercitrin	26.16	346.73	815.51	n.d.
Quercitrin	8.91	52.53	86.79	6.17
Kaempferol‐3‐glucoside	197.07	1093.33	3015.14	6.66
Flavanones				
Naringin	n.d.	n.d.	n.d.	n.d.
Hesperidin	n.d.	n.d.	n.d.	48.92
Anthocyanins				
Cyanidin‐3‐glucoside	n.d.	n.d.	n.d.	n.d.
Delphinidin‐3‐galactoside	n.d.	n.d.	5.13	4.34
Delphinidin 3,5‐diglucoside	22.29	282.79	681.20	n.d.
Petunidin‐3‐glucoside	n.d.	n.d.	2.20	n.d.
Pelargonidin‐3‐glucoside	n.d.	n.d.	n.d.	n.d.
Pelargonidin‐3‐rutinoside	n.d.	n.d.	n.d.	n.d.
Malvidin‐3‐galactoside	n.d.	n.d.	n.d.	n.d.
Dihydrochalcones				
Phloridzin	n.d.	n.d.	n.d.	n.d.
Phloretin	n.d.	n.d.	n.d.	n.d.
Stilbenes				
Resveratrol	n.d.	n.d.	n.d.	n.d.
Total polyphenol content	6552.962	9587.513	20,191.91	14,233.05

*Note:* Values are expressed as mg/kg of dried weight extract.

Abbreviation: n.d., not detected.

The findings obtained are largely consistent with those reported in the literature. In a study conducted by Kikowska and Thiem (2021), *E. campestre* was found to possess a flavonoid‐rich composition, including compounds such as quercitrin, isoquercitrin, rutin, luteolin 7‐O‐β‐d‐glucopyranoside, kaempferol 3‐O‐β‐d‐(2′′‐p‐E‐hydroxycinnamoyl)‐glucopyranoside, and kaempferol 3‐O‐β‐d‐(2′′‐p‐Z‐hydroxycinnamoyl)‐glucopyranoside. In the present study, the detection of kaempferol‐3‐glucoside and its derivatives at high levels supports and confirms these findings.

Similarly, in a study by Al‐Askar et al. (2023), important phenolic compounds, including benzoic acid, catechol, vanillic acid, resveratrol, naringenin, and quercetin, were identified in the plant's methanol extract. Compared with the present study, the detection of vanillic acid and quercetin similarly highlights overlap in the extract composition. The use of multiple solvents during extraction is most likely responsible for detecting distinct chemicals in extracts from the same plant.

### 3.2 Total Phenolic and Flavonoid Content

In recent years, the term “bioactive compounds” has attracted interest for nutraceutical and pharmaceutical applications aimed at improving human health. Among bioactive compounds, phenolics are the most common and exhibit critical health‐promoting properties, including antioxidant, anticancer, and antimicrobial effects (Shao et al. 2025). In this sense, the plant extract's total phenolic content might offer important information about its functional potential. Based on this background, we examined the total phenolic and flavonoid levels in the tested extracts; the results are presented in Table 2. The EA and water extracts had higher total phenolic content than the ethanol and ethanol‐water extracts. However, the ethanol‐water extract had the highest flavonoid content (19.16 mg RE/g), followed by ethanol, water, and EA extracts. These findings indicate that the choice of extraction solvents for *E. campestre* is a critical step in the preparation of functional products. In the literature, several authors have reported different levels of these compounds in members of the *Eryngium* genus, including *E. campestre* . For example, in a recent study by Hawryl et al. (2024), the total phenolic level in the extract of *E. campestre* was found to be 1.171 mg GAE/mL. In another study conducted by Matejić et al. (2018), different extracts of *E. campestre* were examined for the total phenolic and flavonoid contents, and the highest level was obtained with the EA extract (total phenolic content: 111.9 mg GAE/g; total flavonoid content: 164.5 mg QE/g). The total phenolic content varied from 0.2 to 6.1 mg GAE/g in *E. kotschyi* and *E. campestre* var. *virens* root extracts, as reported by Balkan et al. (2020). Azizkhani and Sodanlo (2021), reported a total phenolic content of 120.5 mg GAE/g in the methanol extract of *E. campestre* . The geographical and climatic conditions can explain the differences. Nevertheless, several drawbacks of spectrophotometric techniques for quantifying flavonoids and phenolics exist. For example, AlCl_3_ can form complexes not only with flavonoids but also with other components, thereby affecting the total flavonoid content (Mammen and Daniel 2012). In addition, peptides can reduce the Folin–Ciocalteu reagent (Munteanu and Apetrei 2021). In this sense, spectrophotometric results alone are insufficient to evaluate the content of these bioactive compounds; chromatographic methods are also needed.

**TABLE 2:** Extraction yields, total phenolic and flavonoid content in the tested extracts.

Extracts	Extraction yields (%)	Total phenolic content (mg GAE/g)	Total flavonoid content (mg RE/g)
EA	1.58	32.70 ± 0.28^a^	1.92 ± 0.45^d^
Ethanol	2.54	28.65 ± 0.26^b^	7.62 ± 0.06^b^
Ethanol‐water	6.44	28.67 ± 0.22^b^	19.16 ± 0.30^a^
Water	5.36	32.15 ± 0.12^a^	4.91 ± 0.10^c^

*Note:* Values are reported as mean ± SD of three parallel measurements. Different superscripts indicate significant differences between the tested extracts (*p* < 0.05).

Abbreviations: GAE, gallic acid equivalent; RE, rutin equivalent.

### 3.3 Antioxidant Properties

In the human body, the production of free radicals and their protection, namely the antioxidant system, are in balance. A disruption in this balance can lead to excessive free radical production, a condition known as oxidative stress. In recent times, serious health problems, including cancer, obesity, and diabetes, can result in oxidative stress. In this context, dietary antioxidant intake can be crucial and contribute to the endogenous defense system against free radical attacks. Plants are important natural sources of dietary antioxidants, and several studies indicate their intake can positively affect human health (Zehiroglu and Ozturk Sarikaya 2019). With this in mind, we used a variety of chemical tests, including radical scavenging (ABTS and DPPH), reducing power (CUPRAC and FRAP), phosphomolybdenum, and metal chelation, to investigate the antioxidant effects of *E. campestre* extracts. The results are presented in Table 3. The water extract demonstrated the best ability in the radical scavenging assays (DPPH and ABTS), followed by the ethanol‐water, ethanol, and EA extracts. We use FRAP and CUPRAC tests to measure antioxidants' ability to donate electrons and reduce substances. In the CUPRAC assay, the strongest activity was measured in the ethanol‐water extract (91.19 mg TE/g), followed by the ethanol, water, and EA extracts. Regarding the FRAP assay, the ethanol‐water and water extracts displayed similar abilities (71.66–71.86 mg TE/g), whereas the EA extract showed the weakest ability (50.05 mg TE/g). The phosphomolybdenum assay (PBD) also covers the reduction of Mo(VI) to Mo(V) by antioxidants under acidic conditions and is a popular assay due to its simplicity and low cost (Bibi Sadeer et al. 2020). According to the table, EA extract had the highest PBD assay ability (1.91 mmol TE/g), followed by ethanol, ethanol‐water, and water extracts. Metal chelation testing is a highly significant antioxidant approach for controlling the hydroxyl radical produced in the Fenton reaction. The metal chelating activity of the ethanol‐water and water extracts was nearly the same (34.77–35.58 mg EDTAE/g), but the ethanol extract had the lowest metal chelating ability (14.86 mg EDTAE/g). Numerous research in the literature have demonstrated the antioxidant qualities of *Eryngium* members. For example, Matejić et al. (2018) reported that the water extract of *E. campestre* was the most active in DPPH and ABTS assays, compared with acetone, methanol, and EA extracts. In another study by Balkan et al. (2020), the ethanol extract of *E. campestre* had the strongest ABTS scavenging activity with a value of 1.2 μM TE/g extract. In addition, Azizkhani and Sodanlo (2021) reported the highest DPPH and FRAP activities in the *E. campestre* extract compared with those of *Froriepia subpinnata* and *Mentha spicata* . Several substances in the chemical profiles of the studied extracts can be linked to the found antioxidant activities based on the structure–activity connection, as shown in Table 1. For instance, the EA extract contains more ellagic acid than other extracts, which can be explained by its antioxidant properties (Tošović and Bren 2020; García‐Niño and Zazueta 2015).

**TABLE 3:** Antioxidant properties of the tested extracts.

Extracts	DPPH (mg TE/g)	ABTS (mg TE/g)	CUPRAC (mg TE/g)	FRAP (mg TE/g)	MCA (mg EDTAE/g)	PBD (mmol TE/g)
EA	11.30 ± 0.41^d^	77.76 ± 2.65^c^	79.66 ± 3.41^b^	50.05 ± 2.50^c^	19.03 ± 0.94^b^	1.91 ± 0.13^a^
Ethanol	13.82 ± 0.89^c^	94.37 ± 3.67^b^	89.07 ± 2.88^a^	58.31 ± 0.27^b^	14.86 ± 1.20^c^	1.66 ± 0.10^b^
Ethanol‐water	37.07 ± 0.71^b^	171.87 ± 2.64^a^	91.19 ± 0.60^a^	71.66 ± 1.40^a^	34.77 ± 0.08^a^	0.73 ± 0.03^c^
Water	42.34 ± 1.20^a^	176.11 ± 1.60^a^	86.91 ± 1.70^a^	71.86 ± 1.73^a^	35.58 ± 0.37^a^	0.55 ± 0.01^d^

*Note:* Values are reported as mean ± SD of three parallel measurements. Different superscripts indicate significant differences between the tested extracts (*p* < 0.05).

Abbreviations: EDTAE, EDTA equivalent; MCA, metal chelating activity; PBD, phosphomolybdenum; TE, trolox equivalent.

### 3.4 Enzyme Inhibitory Effects

Research into solutions for diseases that are becoming increasingly prevalent in today's scientific world is gaining momentum. Enzymes act as versatile agents in these solutions. Inhibiting certain key enzymes is an effective way to treat these diseases—a phenomenon known as enzyme inhibition theory (Singh et al. 2024). For instance, inhibiting the amylase enzyme offers diabetics an alternative approach to controlling blood glucose levels (Kaur et al. 2025). Similarly, inhibiting the tyrosinase enzyme is an important strategy for treating pigmentation disorders. Numerous compounds have been designed synthetically as enzyme inhibitors for this purpose, but concerns about their use have highlighted the need to replace them with natural, safe, and effective alternatives. With this in mind, we examined the enzyme inhibitory effects of the tested extracts against cholinesterase, amylase, glucosidase, and tyrosinase. The results are shown in Table 4. With values of 2.68 and 3.17 mg GALAE/g, respectively, ethanol extract showed the greatest inhibition of AChE and BChE. Regarding tyrosinase inhibition, the strongest inhibition was detected in the ethanol extract (76.39 mg KAE/g), followed by EA, ethanol‐water, and water extracts. Among the extracts, ethanol and ethanol‐water extracts exhibited almost identical glucosidase inhibitory effects, whereas the water extract showed the weakest effect (0.03 mmol ACAE/g). The studied extracts' amylase inhibiting effects were arranged in decreasing order: EA>ethanol>ethanol‐water>water.

**TABLE 4:** Enzyme inhibitory effects of the tested extracts.

Extracts	AChE (mg GALAE/g)	BChE (mg GALAE/g)	Tyrosinase (mg KAE/g)	Amylase (mmol ACAE/g)	Glucosidase (mmol ACAE/g)
EA	2.29 ± 0.32^a^	2.87 ± 0.55^a^	70.45 ± 1.28^a^	0.62 ± 0.01^a^	0.72 ± 0.04^b^
Ethanol	2.68 ± 0.06^a^	3.17 ± 0.30^a^	76.39 ± 5.17^a^	0.51 ± 0.01^b^	1.00 ± 0.02^a^
Ethanol‐water	2.34 ± 0.03^a^	1.30 ± 0.07^c^	50.30 ± 1.35^b^	0.36 ± 0.01^c^	1.00 ± 0.14^a^
Water	0.36 ± 0.01^b^	2.51 ± 0.06^b^	3.17 ± 0.57^c^	0.05 ± 0.01^d^	0.03 ± 0.01^c^

*Note:* Values are reported as mean ± SD of three parallel measurements. Different superscripts indicate significant differences between the tested extracts (*p* < 0.05).

Abbreviations: ACAE, acarbose equivalent; GALAE, galantamine equivalent; KAE, kojic acid equivalent; na, not active.

In the literature, there is no report on the enzyme‐inhibitory effects of *E. campestre* . The presence of certain chemicals in the extracts may be responsible for the reported enzyme‐inhibitory effect on the structure–activity connection. For example, chlorogenic acid (Oh et al. 2019; Zheng et al. 2020; Oboh et al. 2013), rutin (Dubey et al. 2017; Si et al. 2012), quercetin (Huang et al. 2024; Liao et al. 2022) or ellagic acid (Oh et al. 2021) are already known as enzyme inhibitors. For example, Işık and Beydemir (2021) reported that the hydroxyl groups in the chlorogenic structure are able to attach to a site located outside the active region of AChE, which in turn suppresses the enzyme's activity. In a previous study by Shojazadeh et al. (2023), chlorogenic acid appears to inhibit tyrosinase primarily through reversible, competitive binding at or near the enzyme's active site, with this interaction stabilized by hydrogen bonds and hydrophobic interactions. Chlorogenic acid engaged with the catalytic residues located in the amylase active site and additionally associated, via hydrogen bonding and hydrophobic contacts, with specific amino acid residues positioned away from the active site, thereby displaying mixed‐type inhibition (Zheng et al. 2020). Similar to chlorogenic acid, both the number and the specific position of hydroxyl groups on phenolic or flavonoid rings can influence how these compounds interact with the active or allosteric sites of the enzymes under investigation. Furthermore, the inherently complex nature of phytochemicals (including non‐phenolic constituents) and their mutual interactions—whether synergistic or antagonistic—can modulate their enzyme inhibitory effects. At this stage, further studies focusing on the isolation and characterization of individual compounds are required to determine which compound(s) are responsible for the observed enzyme inhibitory activities.

### 3.5 Anticancer Activity of *E. campestre*

Breast and cervical cancers are among the most common malignancies affecting women and represent a significant global health threat. Breast cancer is the most common kind of cancer diagnosed in women, making up around one‐third of all cancer cases (Sung et al. 2021). Additionally, cervical cancer is the seventh most frequent disease worldwide and the fourth most common cancer in women (Momenimovahed and Salehiniya 2017). Although cancer treatments generally rely on methods such as chemotherapy and radiotherapy, these approaches can lead to adverse effects due to their potential to damage healthy cells. For this reason, researchers have focused on plants with various biological activities as alternative or complementary therapeutic agents to overcome these complications (Ocal et al. 2022).

**FIGURE 1:**
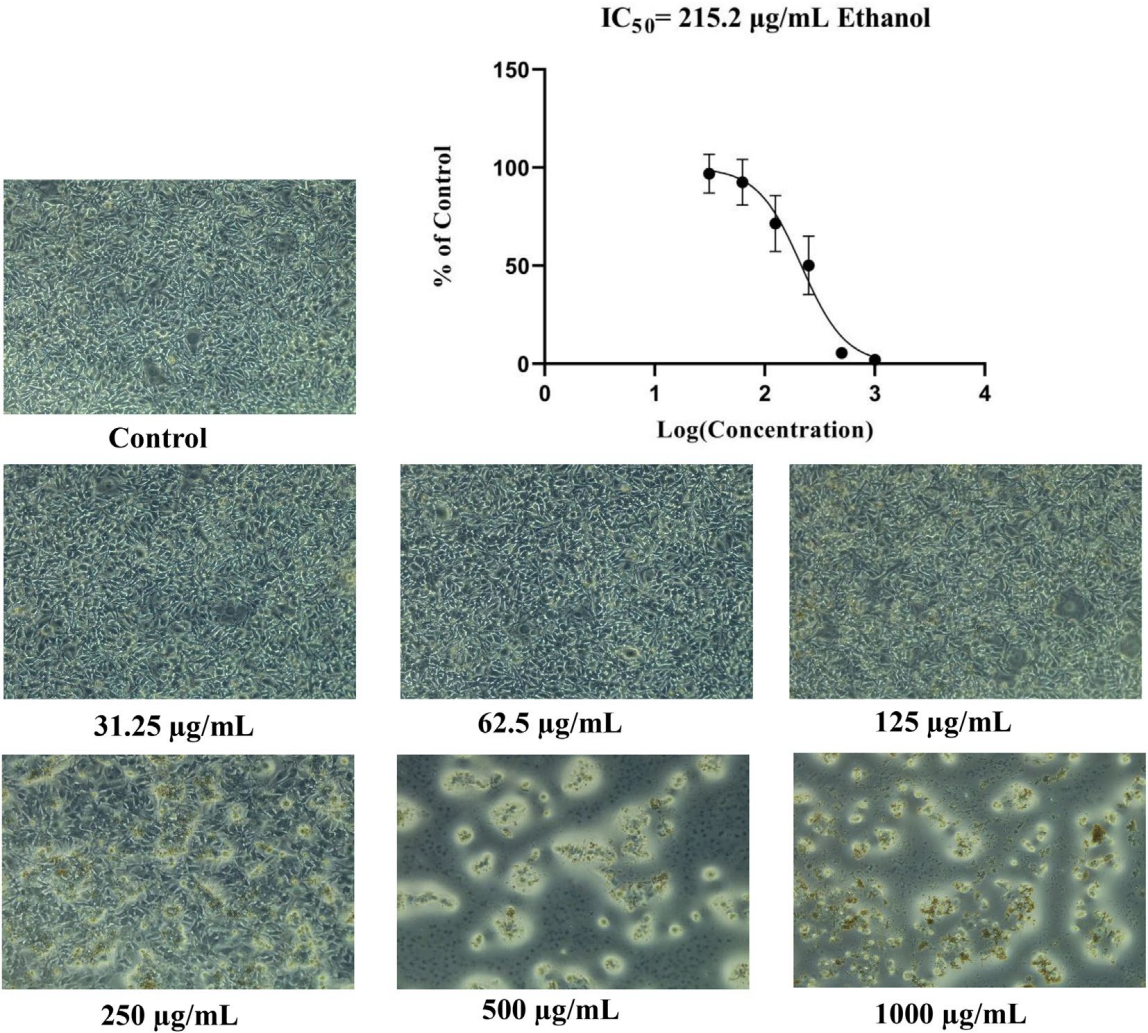
IC_50_ value and microscopic view of cell viability of the ethanol extract on the MDA‐MB‐231 cell line.

In this study, the anticancer activity of *E. campestre* extracts was evaluated against the MDA‐MB‐231 and HeLa cell lines. The analysis revealed that the lowest IC_50_ value was 215.2 μg/mL for the ethanol extract in the MDA‐MB‐231 cell line (Figure 1).

**FIGURE 2:**
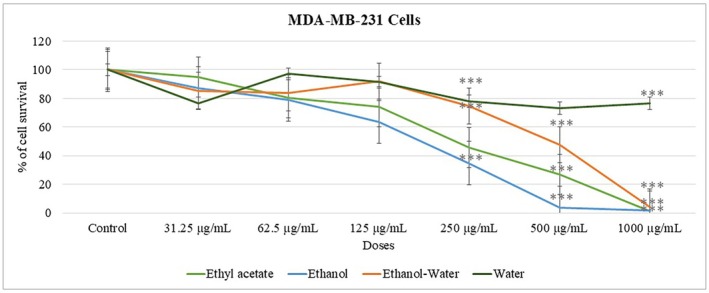
Percentage of cell viability of the MDA‐MB‐231 cell line at different concentrations after 48 h. Data are presented as mean ± standard error. *p < 0.05, **p < 0.01, ***p < 0.001.

The most pronounced reduction in cell viability was measured with the EA extract at 1000 μg/mL, with a viability rate of 1.03%. Similarly, at 1000 μg/mL, the ethanol extract and the ethanol–water extract displayed cell viabilities of 1.54% and 4.26%, respectively. In addition, treatment with 500 μg/mL of the ethanol extract resulted in a cell viability of 3.61% (Figure 2) (Figure S1).

These findings are consistent with previous reports on other *Eryngium* species. For instance, the aerial parts of *E. thyrsoideum* have been notified to exhibit strong anticancer activity against MDA‐MB‐231 cells (Hosseini et al. 2022). In a previous study, the anticancer activity of the aerial parts of *E. kotschyi* was evaluated against the MDA‐MB‐231 cell line (Arslan et al. 2022), and consistent with the present findings, the EA extract showed the most potent cytotoxicity.

Furthermore, studies on breast cancer cells have reported that ferulic acid suppresses epithelial–mesenchymal transition (EMT), thereby reducing the metastatic potential of cancer cells and inhibiting their migration and invasion capacities in a dosage‐dependent manner (Zhang et al. 2016). In light of these findings, the high levels of ferulic acid detected in *E. campestre* plant extracts suggest that this compound may act as a potential anti‐metastatic agent.

**FIGURE 3:**
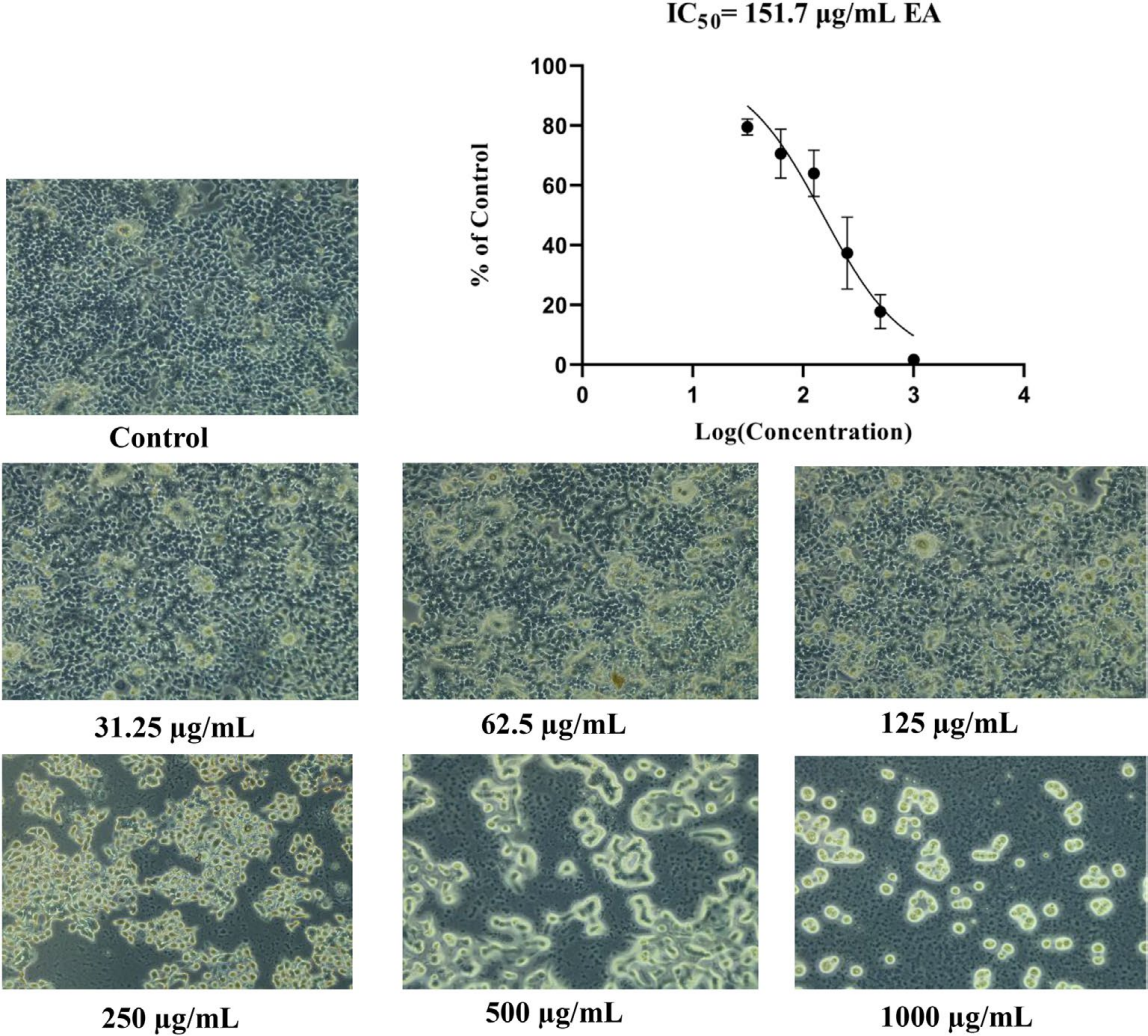
IC_50_ value and microscopic view of cell viability of the Ethyl acetate extract on the HeLa cell line.

**FIGURE 4:**
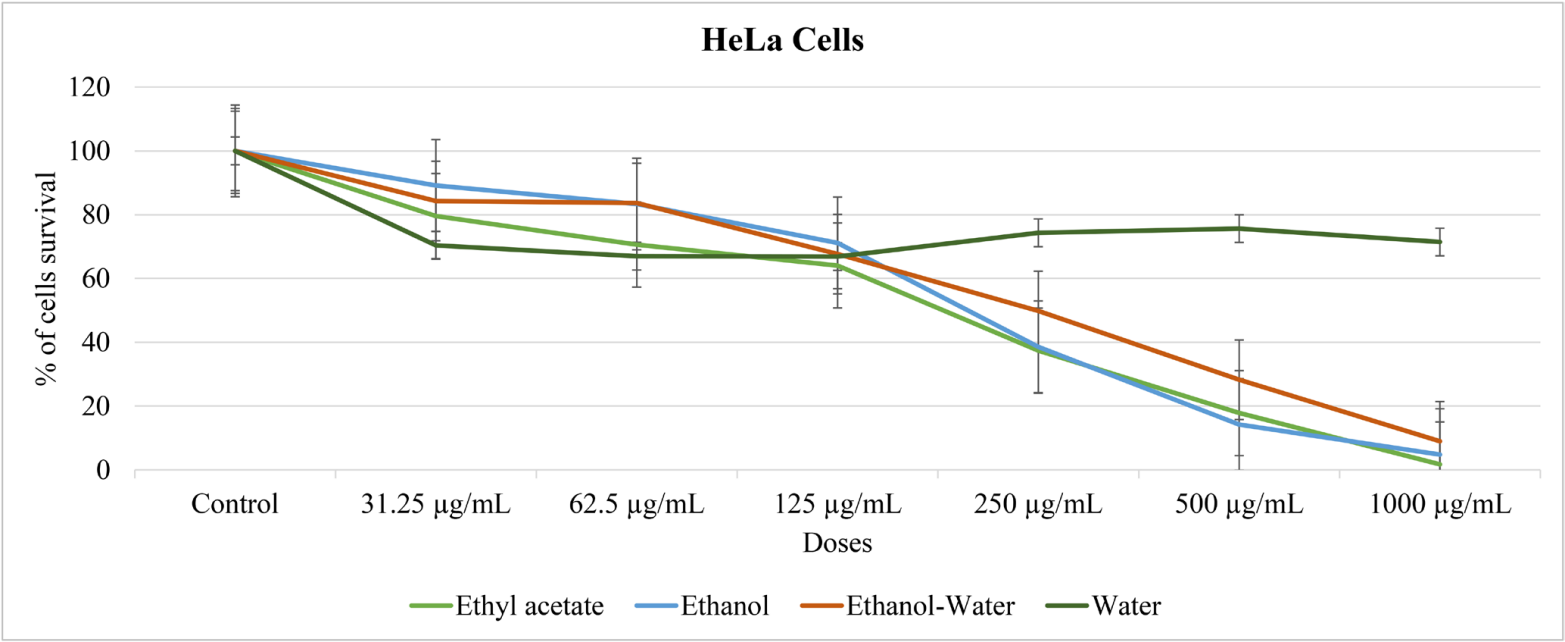
Percentage of cell viability of the HeLa cell line at different concentrations after 48 h.

Upon investigating the anticancer activity of *E. campestre* plant extracts on the HeLa cell line, the lowest IC_50_ value was observed for the EA extract, at 151.7 μg/mL (Figure 3). With a viability of 1.72% at 1000 μg/mL, the EA extract caused the greatest reduction in HeLa cell viability. In the ethanol extract, cell viability was 4.78% at 1000 μg/mL, while in the ethanol–water extract, 8.96% of the cells remained viable (Figure 4) (Figure S1).

The more pronounced decrease in cell viability observed in EA, ethanol, and ethanol‐water extracts compared to the water extract in MDA‐MB‐231 and HeLa cell lines may be attributed to the bioactive compounds contained in these extracts. The fact that these three extracts, in particular, contain isoquercitrin can be considered one of the factors contributing to the observed cytotoxic effect. Studies have shown that isoquercitrin induces apoptosis in various cancer cell lines, promoting cell death, and exhibits cytotoxic activity. Furthermore, isoquercitrin has been shown to regulate various signaling pathways implicated in cancer development, including the PI3K/Akt pathway (Song et al. 2025; Wu et al. 2017).

Moreover, it was determined that these three extracts, in which anticancer effects were observed, also contained the compound delphinidin‐3,5‐diglucoside. The literature reports that delphinidin and its derivatives inhibit cell growth in different molecular subtypes of cancer cell lines and can exhibit anticancer potential by inducing apoptosis. Additionally, it has been suggested that the anticancer effect of delphinidin may be related to Nrf2 activation and NF‐κB suppression (Iqbal et al. 2024; Xu et al. 2020). Therefore, it is thought that the anticancer effect observed in EA, ethanol, and ethanol‐water extracts may be due to the bioactive compounds they contain.

Studies have demonstrated the action of *Eryngium* species on the HeLa cell line. Ethanol extracts obtained from the leaves, stems, roots, and whole plant of *E. creticum* were investigated for their cytotoxic and antiproliferative activities against cervical cancer cells. The results showed that the ethanol extracts exhibited dose‐ and time‐dependent cytotoxicity against the HeLa cell line. Among the studied extracts, the ethanol extract from leaves showed a powerful effect, with an IC_50_ value ≤ 47.24 μg/mL (Dirani et al. 2014). Ethanol extracts are known to be rich in secondary metabolites, and their concentrations vary among the root, stem, and leaf parts of the plant. Accordingly, the most potent effect was observed in the ethanol leaf extract. Consistent with these findings, our study also revealed that the ethanol extract exhibited one of the strongest activities on the tested cell lines.

Furthermore, ferulic acid has been shown to inhibit the proliferation of cervical cancer cells (Gao et al. 2018). Likewise, one of the main substances identified in our investigation, vanillic acid, has been observed to stimulate apoptosis in tumor cells, suggesting that it may possess anti‐tumor properties (Gong et al. 2019). In a study on the HeLa cell line, ellagic acid was reported to reduce cell proliferation in a dose‐dependent manner, induce apoptosis, and inhibit cell growth by causing cell cycle arrest at the G2 phase. In the in vivo model, it significantly reduced tumor volume and weight, increased apoptotic activity, and exhibited a strong antitumor effect without observable toxicity (Xia et al. 2020). Previous studies have demonstrated that ellagic acid exhibits significant anticancer activity in MDA‐MB‐231 breast cancer cells. It has been reported that ellagic acid inhibits cell proliferation in a dose‐dependent manner and reduces cell viability in both cisplatin‐sensitive and cisplatin‐resistant cells. Mechanistically, ellagic acid may reduce chemoresistance, suppress tumor cell invasion and metastasis, inhibit angiogenesis, and induce apoptosis. Collectively, these findings suggest that ellagic acid exerts multiple anticancer effects in MDA‐MB‐231 breast cancer cells (Saltoglu et al. 2026). Ellagic acid exhibited dose‐dependent cytotoxic activity in the HeLa and MDA‐MB‐231 cell lines, significantly reducing cell viability in both models. In MDA‐MB‐231 cells, this effect was associated with cell cycle regulation mediated by CDK6 (cyclin‐dependent kinase 6), whereas in HeLa cells, ellagic acid was reported to inhibit proliferation by inducing apoptosis through mitochondrial and cell cycle–related mechanisms (Shihab et al. 2026). Rutin has been reported to reduce cell viability in a dose‐dependent manner, suppress proliferation, and induce apoptosis in cervical cancer cells. These effects have been associated with the downregulation of Jab1 (Jun activation domain‐binding protein 1) and Bcl‐2 (B‐cell lymphoma 2), along with upregulation of p53 (tumor protein p53) and Bax (Bcl‐2‐associated X protein). In addition, it has been shown to disrupt mitochondrial membrane potential, triggering cytochrome c release and activation of caspase‐3/caspase‐9, while also increasing intracellular reactive oxygen species (ROS) levels, thereby promoting apoptotic processes (Pandey et al. 2021). This observation may be based on differences in the phytochemical composition of the extracts, variations in bioactive compound concentrations, and differences in extraction efficiency, which may have resulted in lower levels of active constituents in some samples. Overall, these findings are consistent with the literature, which suggests that these compounds exert multiple anticancer effects primarily through inhibition of cell proliferation and induction of apoptosis. In agreement with previous reports, the cytotoxic activity observed in the present study appears to be associated with the presence of ellagic acid, rutin, and other phenolic compounds identified in the ethyl acetate and ethanol extracts. The pronounced reduction in cell viability exhibited by these extracts further supports the potential contribution of these constituents to the observed anticancer activity. Nevertheless, given the complex phytochemical composition of plant extracts, the biological effects cannot be attributed exclusively to these compounds. Therefore, the observed cytotoxic activity is likely due to both the individual effects of these phenolics and potential synergistic interactions among multiple bioactive constituents.

In conclusion, *E. campestre* extracts demonstrated significant cytotoxic and antiproliferative effects against both MDA‐MB‐231 and HeLa cell lines. In particular, the low IC_50_ values obtained for the EA and ethanol extracts suggest that these solvents effectively concentrated bioactive constituents. The observed anticancer activity may be associated with the presence of phenolic compounds identified in these extracts, including ellagic acid, rutin, ferulic acid, vanillic acid, and kaempferol derivatives. Nevertheless, the contribution of synergistic interactions among multiple phytochemicals should also be considered. These findings highlight the potential of *E. campestre* as a source of bioactive compounds for further preclinical investigations toward the development of novel anticancer agents.

### 3.6 Antimicrobial Activity of *E. campestre* Extracts

Antibiotics play a major role in modern medicine; their use has reduced childhood mortality and increased life expectancy. Additionally, they are necessary for treatments like chemotherapy and invasive surgery. However, as the prevalence of diseases caused by drug‐resistant bacteria increases globally, the risk of incurable illnesses is increasing. There is a dearth of novel antibiotics in development, alongside a rise in resistance to current treatments (Blair et al. 2015). For these reasons, antimicrobial compounds derived from plant extracts are considered promising alternative treatment options against resistant strains, and research in this field is gaining increasing importance.

**FIGURE 5:**
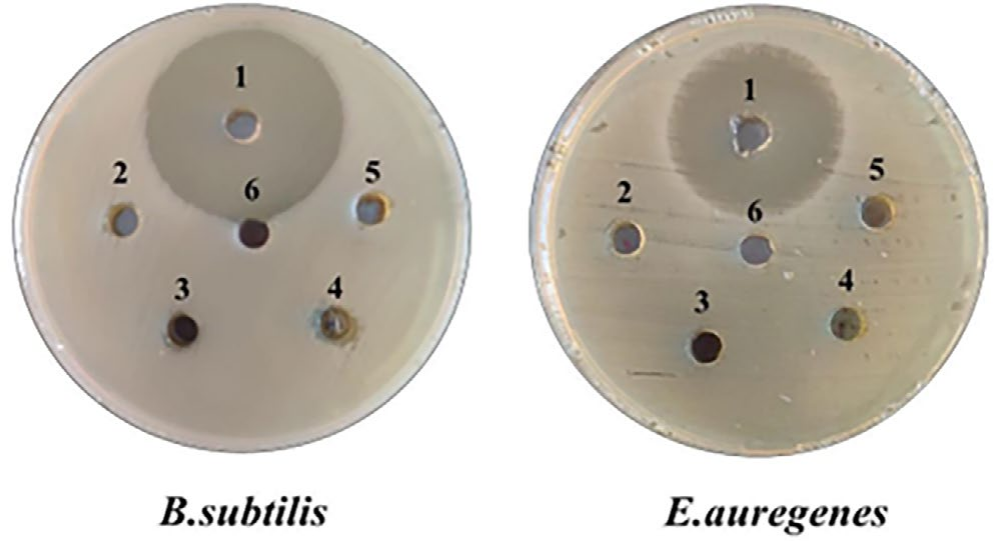
Representation of the antimicrobial activity of the extracts against *B. subtilis* and *E. aerogenes* (1: Positive control, 2: Negative control, 3: Ethyl acetate, 4: Ethanol, 5: Ethanol‐water, 6: Water).

This study employed the agar well diffusion method to evaluate the antibacterial activity of *E. campestre* . At the tested concentrations, no inhibition zones were observed for any bacterial strain, except for the positive control. These results demonstrate that the extracts exhibited no measurable antibacterial activity against the tested microorganisms under the applied experimental conditions (Figure 5).

In a previous study, the antibacterial effect of ethanol extracts from the roots and shoots of *E. campestre* at 100 mg/mL was investigated, and weak activity was observed against various bacterial strains. In the current study, the lack of antimicrobial activity is attributed to differences in the applied concentrations. Furthermore, this inconsistency is attributed to the variable antimicrobial activity across plant parts, including roots, stems, shoots, leaves, and fruits (Zaki et al. 2025).

In the literature, ellagic acid has been reported to exhibit selective antibacterial activity against certain bacteria, attributed to its interactions with bacterial enzymes and proteins, thereby inhibiting their functions (Pinho et al. 2014). Likewise, rutin and other polyphenols (hesperetin, hesperidin, ferulic acid, quercetin derivatives, and isoquercitrin) have been reported to inhibit the growth of antibiotic‐resistant bacteria, reduce biofilm formation, and suppress virulence and quorum sensing mechanisms. However, in the present study, no significant antibacterial or antibiofilm activity was observed for any of the tested polyphenols, including rutin. This discrepancy may be attributed to strain‐dependent susceptibility, insufficient effective concentrations, and methodological variations, as the antimicrobial activity of polyphenols has been reported to vary considerably across microbial models (Ivanov et al. 2022). Although ellagic acid, rutin, and other polyphenols have been reported to exhibit antimicrobial activity, no similar effect was observed in the present study. This discrepancy may be attributed to strain‐dependent differences in susceptibility, insufficient concentrations of the active compounds in the extracts, or methodological variations.

### 3.7 Anti‐Quorum Sensing Activity Assay

**FIGURE 6:**
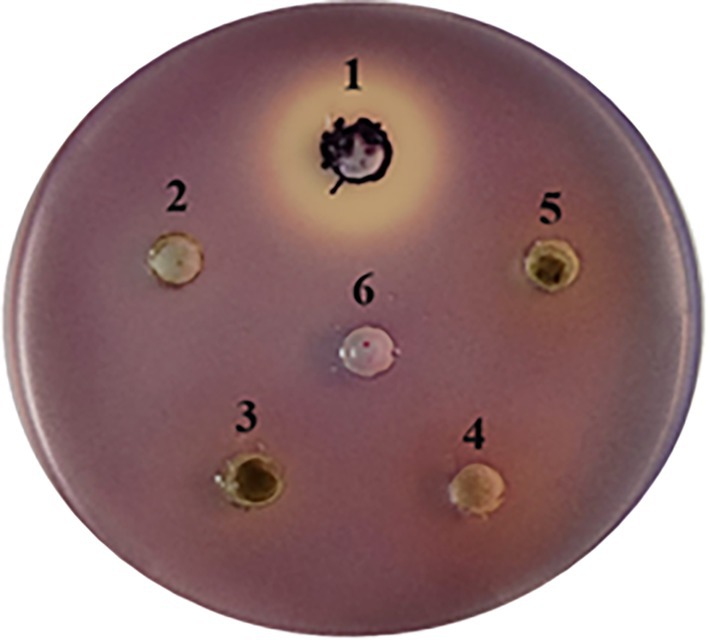
Potential of the extracts to inhibit violacein production in *C. violaceum* CV026 (1: Positive control ( *C. violaceum* 12472), 2: Negative control (water), 3: Ethyl acetate, 4: Ethanol, 5: Ethanol‐water, 6: Water).

Quorum sensing (QS) and QS‐targeting anti‐QS compounds are considered alternative therapeutic agents to antibiotics due to their capacity to suppress pathogenicity (Tüfekci et al. 2020). Therefore, the anti‐QS activity of *E. campestre* was tested; however, no effect on the positive control was observed at the concentrations studied (Figure 6).

Violacein pigment production in *C. violaceum* ATCC 12472 is generally regulated by long‐chain AHL molecules (C10–C16). The lack of a significant inhibitory effect of the extract on violacein production in our study may be due to the compounds in the extract affecting short‐chain AHL molecules (Tüfekci et al. 2020).

The geographical location, collection season, and extraction technique all have significant roles in identifying the chemical composition of the resulting extract. These factors collectively influence the qualitative and quantitative features of bioactive compounds, which, in turn, directly affect the level and spectrum of antimicrobial activity displayed by the extract. The absence of any inhibition of violacein production at the tested concentrations in the current study may be due to the following reasons.

## 4 Conclusion

The greatest total polyphenol substance was observed in the ethanol–water extract. Especially, 3‐ and 4‐hydroxybenzoic acids, vanillic acid, p‐coumaric acid, and ferulic acid were consistently abundant across all extracts. The EA and water extracts exhibited much more total phenolic content than the ethanol and ethanol‐water extracts, whereas the ethanol‐water extract showed the highest flavonoid content. Moreover, according to the literature, the enzyme‐inhibitory effects of *E. campestre* have not been previously reported. Certain phytochemicals in the extracts may account for the determined inhibitory action, suggesting a potential structure–activity relationship. In this regard, the study provides the first evidence highlighting the enzyme‐inhibitory potential of *E. campestre* .

Besides, the antimicrobial and anti‐quorum sensing properties of *E. campestre* extracts were assessed in this investigation; however, no significant activity was observed at the tested concentrations. The findings suggest that new experimental designs using different extraction methods, fractionation techniques, and higher concentrations are needed to further evaluate the antimicrobial potential of these plant extracts. Additionally, purification at the compound level would allow a more accurate assessment of the anti‐QS potential.

Moreover, the anticancer activity of *E. campestre* extracts was evaluated. The lowest IC_50_ value for the MDA‐MB‐231 cell line was 215.2 μg/mL for the ethanol extract, whereas for the HeLa cell line, it was 151.7 μg/mL for the EA extract. These results indicate a moderate cytotoxic effect of the extracts against the tested cancer cell lines. Overall, *E. campestre* may represent a promising source of bioactive compounds for further pharmacological investigations. However, additional in vitro and in vivo studies, as well as compound‐level isolation and mechanistic analyses, are necessary to better elucidate its anticancer potential.

## Author Contributions

**Pınar Baloglu:** writing – original draft, writing – review and editing. **Gamze Akıllı:** investigation, writing – original draft, writing – review and editing, methodology. **Mehmet Cengiz Baloglu:** validation, writing – review and editing, methodology. **Ayşenur Özdemir Kanat:** writing – original draft, writing – review and editing. **Muammer Bahsi:** writing – review and editing, investigation. **Nurdan Tugba Iscen:** writing – original draft, writing – review and editing. **Yasemin Celik Altunoglu:** writing – original draft, writing – review and editing, investigation, methodology. **Massimo Ricciutelli:** writing – review and editing, investigation. **Giovanni Caprioli:** writing – review and editing, investigation. **Kübra Seyhan:** writing – original draft, investigation, methodology, writing – review and editing. **Gökhan Zengin:** writing – original draft, writing – review and editing, investigation, methodology. **Agnese Santanatoglia:** investigation, writing – review and editing. **Milena Terzic:** writing – review and editing, investigation.

## Funding

The authors have nothing to report.

## Conflicts of Interest

The authors declare no conflicts of interest.

## Supporting information


**Figure S1:** IC_50_ values and microscopic view of cell viability of the extracts on MDA‐MB‐231 and HeLa cell lines.

## Data Availability

Data are available from the corresponding author upon reasonable request.
