# Supplementary material for: Potent Therapeutic Insights From Eryngium campestre: Phytochemical Profiling and In Vitro Evaluation
Source: Food Sci Nutr. 2026 Aug 10;14(8):e72250. doi: 10.1002/fsn3.72250 (PMC13458033; doi:10.1002/fsn3.72250)

MDA-MB-231 cell line

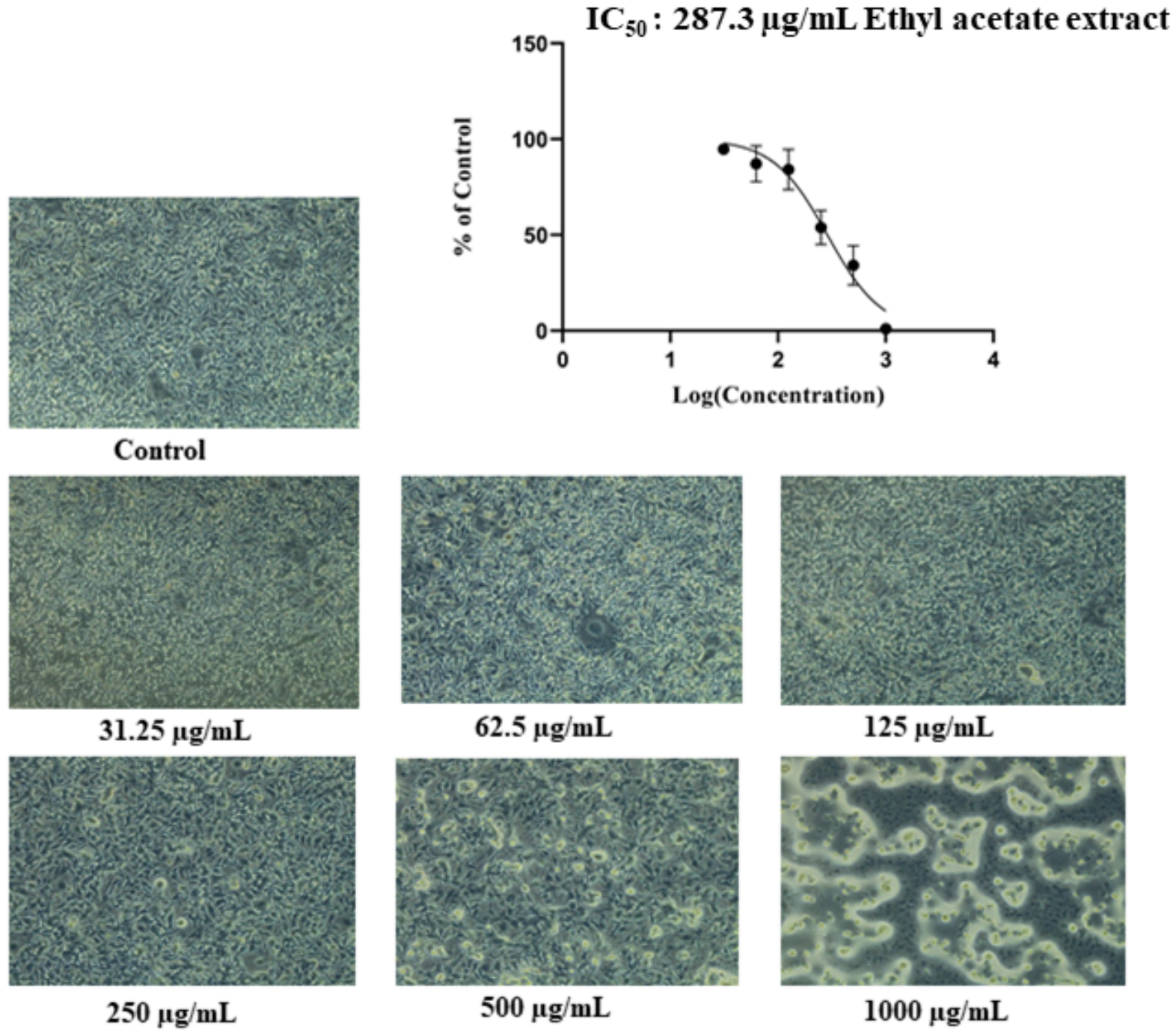

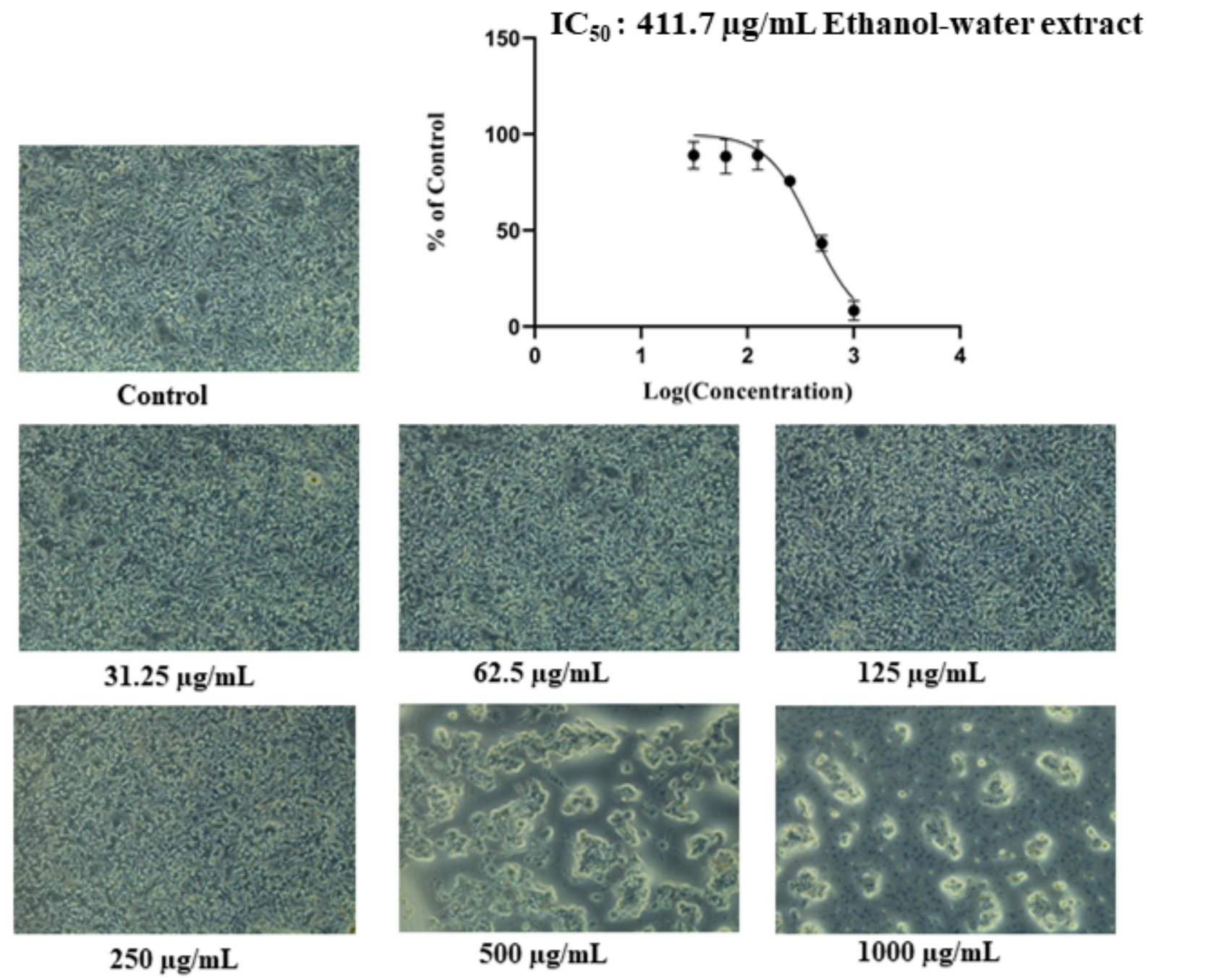

HeLa cell line

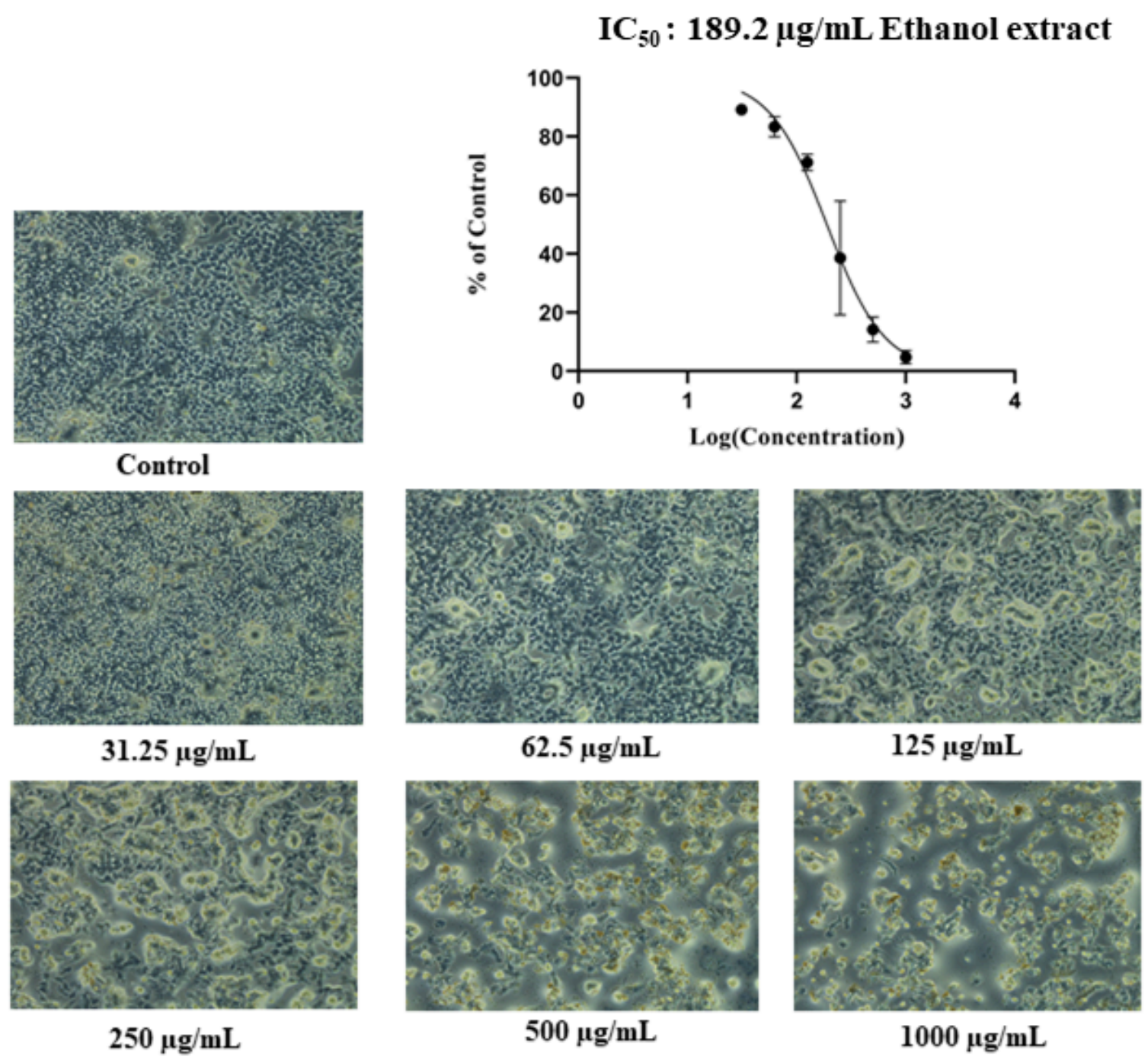

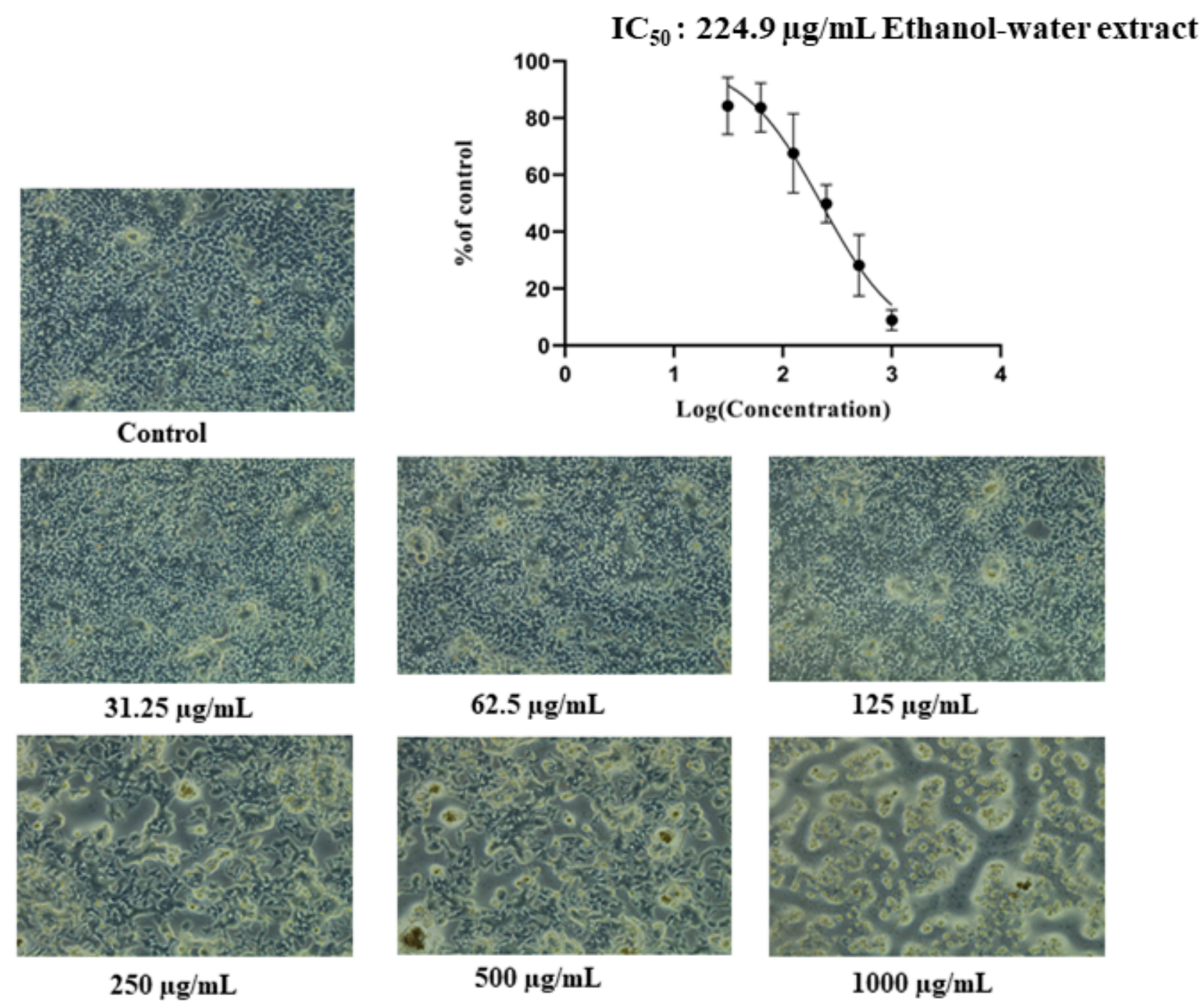

Supplement: Supplementary file 1 [file FSN3-14-e72250-s001.pdf]
